# White agrocybe cylindracea outperforms the brown strain in nutritional value, flavor profile, and anticancer activity

**DOI:** 10.1016/j.fochx.2026.104579

**Published:** 2026-10-05

**Authors:** Chengzhen Gu, Yangzheng Lin, Mengmeng Hao, Zhiying Lin, Jiaqi Fang, Jianwen Luo, Shujing Sun

**Affiliations:** College of Life Sciences, Fujian Agriculture and Forestry University, Fuzhou 350002, China

**Keywords:** *A**. cylindracea***, Nutritional components, Biological activity, Quality, Volatile compounds

## Abstract

*Agrocybe cylindracea*, an edible and medicinal mushroom, comprises white (BC) and brown (CS) strains. The white strain is a new cultivar whose nutritional value remains unexplored. Nutritional components and bioactivities of BC and CS were compared using electronic nose, electronic tongue, texture analyzer, chromatography, taste re-addition experiments, and cytotoxicity assays. BC showed significantly higher crude polysaccharides, soluble protein, crude fiber, equivalent umami concentration, taste-active compounds (free amino acids and 5′-nucleotides), and stronger sourness, bitterness, sweetness, and umami than CS. Aspartic acid, glutamine, tryptophan, lysine, and 5′-cytidylic acid mainly contributed to sourness; glutamine and tryptophan to umami. Gas Chromatography-Mass Spectrometry (GC–MS) identified 41 (BC) and 37 (CS) volatiles with distinct aroma profiles. Both strains inhibited HCT116 and HGC27 proliferation via elevated reactive oxygen species (ROS). This first systematic comparison demonstrates BC exceeds CS in nutritional value, flavor, and anticancer activity.

## 1 Introduction

Edible mushrooms represent an important food and medicinal resource in the global agricultural economy, with annual worldwide production exceeding 50 million tons (Bisht & Negi, 2026; Cao & Yuan, 2025; Dong et al., 2022; Li et al., 2023). They are rich in polysaccharides, proteins, free amino acids, vitamins, and minerals, as well as bioactive compounds such as polyphenols and terpenoids that exhibit anti-inflammatory, anti-tumor, and antioxidant properties (Hamza et al., 2024; Mishra & Shankar, 2025). These nutritional and medicinal attributes directly determine mushroom quality. However, despite growing consumer demand for superior nutrition amid rising living standards, the quality of many edible mushrooms remains insufficiently characterized.

Nutrients in edible mushrooms encompass volatile and non-volatile constituents. The non-volatile fraction—comprising polysaccharides, proteins, free amino acids, 5′-nucleotides, and organic acids—constitutes the core nutritional matrix. Polysaccharides, as the principal bioactive components, have been extensively investigated regarding extraction optimization, structural elucidation, and bioactivity verification (Yang et al., 2019). Proteins and free amino acids are essential nutritional elements, with the latter categorized into umami, sweet, bitter, and aromatic types (Wang et al., 2020). Principal component analysis and taste activity values have identified glutamic acid, malic acid, alanine, proline, leucine, and aspartic acid as the characteristic flavor compounds of *Agaricus bisporus* fruiting bodies across different growth stages (Wang et al., 2018). Enzymatic degradation of nucleic acids yields various nucleotides, whose synergistic interaction with flavor amino acids imparts distinct taste profiles to mushrooms (Sun et al., 2020) . The principal volatile compounds of edible mushrooms include eight‑carbon compounds, aldehydes, alcohols, esters, ketones, sulfur-containing compounds, and acid derivatives (Yu et al., 2024). (Xu et al., 2023) Xu et al. employed HS-SPME-GC–MS to analyze volatile components of *Lepista sordida* mycelia and fruiting bodies, detecting 116 compounds in total; 40 were shared between both tissues, including 1-octen-3-ol, trans-nerolidol, and benzaldehyde. Relative odor activity value analysis further identified n-valeric acid, citral, and 1-octen-3-ol as characteristic aroma compounds of fruiting bodies, and γ-nonalactone as that of mycelia. Nevertheless, systematic studies on the nutritional composition and biological activities of edible mushrooms remain limited.

*A. cylindracea* is widely distributed across northern temperate and subtropical regions, though rare in tropical areas. In China, it occurs primarily in Guangdong, Yunnan, Zhejiang, Fujian, and Jiangxi provinces (Yang et al., 2024). As an edible and medicinal mushroom, its fruiting bodies are rich in polysaccharides, high-quality proteins, and various amino acids. Pharmacologically, it exhibits therapeutic effects including strengthening the spleen, clearing heat and removing dampness, and anti-tumor activity (El-Maradny et al., 2021; Jing et al., 2018; Liu et al., 2020). It is considered an ideal dietary option for patients with hypertension, cardiovascular diseases, and obesity, as well as for health-conscious consumers. *A. cylindracea* is a major edible and medicinal mushroom, consumed directly and widely used in condiments (e.g., mushroom sauce) and instant soup bases. It comprises two main strains: white and brown. The brown strain dominates both cultivation and the commercial market, whereas the white strain is a newly recognized variety (2022) that has only recently achieved industrial cultivation and has low market acceptance. A systematic quality evaluation of this new white strain is therefore timely and necessary. Previous studies on the white strain were limited to protein and amino acid contents, leaving its quality, flavor, and bioactivity largely uncharacterized (Lin, 2026). Although the nutritional properties and antitumor activity of brown strain have been extensively studied, differences between white and brown strains in these key quality dimensions had not been systematically reported.

In this study, an electronic nose, electronic tongue, texture analyzer, chromatographic techniques, re-addition experiments of taste-active substances, and cytotoxicity assays were used to comprehensively analyze the nutritional components (polysaccharides, proteins, amino acids, nucleotides, crude fiber, and volatile compounds) and antitumor activity of the white and brown strains. Polysaccharides are recognized immunological components, protein content reflects nutritional value, and crude fiber is associated with intestinal health; together, these indicators reflect the nutritional quality and health-promoting functional properties of *A. cylindracea*. This study provides the first parallel comparison of the nutritional components, flavor characteristics, and antitumor activity of white and brown strains, supporting the quality evaluation and industrial promotion of the white strain. The results will fill the data gap in strain-level quality comparisons and provide direct evidence for assessing the germplasm value of this newly recognized variety. Promoting the white strain addresses the structural homogeneity of cultivated *A. cylindracea* and reduces the industry's dependence on a single strain, representing a shift from monoculture toward strain diversification. The white strain is not a replacement for the brown strain but a structural supplement to the cultivated strain pool and germplasm resource bank. This study will provide scientific evidence for why such a supplement is worth promoting.

## 2 Material and methods

### 2.1 Samples

This study employed two *A. cylindracea* strains: the white Baicha No. 1 (BC) and the brown Gucha No. 2 (CS). BC and CS mycelia were inoculated into mushroom bags and incubated indoors at 20–22 °C. Once fully colonized, the bags were transferred to a fruiting chamber at 25 °C. After 7 days of maturation, the bags were opened when primordia emerged. The primordia were grown for 2 days to the mature stage, harvested, and stored at −80 °C. Human colon cancer (HCT116) and gastric cancer (HGC27) cell lines were obtained from Shanghai Zhongqiao Xinzhou Biotechnology Co., Ltd. Standards of 20 amino acids and six 5′-nucleotides (HPLC ≥98%) were obtained from Beijing Solarbio Science & Technology Co., Ltd.

### 2.2 Instruments

Intelligent mushroom fruiting chamber (ZTKX-CG-1200, Beijing Zhitai Kangxing Biotechnology Co., Ltd.), biochemical incubator (SPX—250B—Z, Shanghai Boxun Industrial Co., Ltd.), benchtop high-speed refrigerated centrifuge (H1850R, Hunan Xiangyi Laboratory Instrument Development Co., Ltd.), microplate reader (Varioskan LUX, Thermo Fisher Scientific). Texture analyzer (TMS-Touch, Food Technology Corporation). Crude polysaccharides and soluble proteins were measured by UV–Vis spectrophotometer (UV-1801, Beijing Beifen-Ruili Analytical Instrument Co., Ltd.). Taste profile was determined by electronic tongue (iTongue30, THINKSENSO & SENSO). Free amino acid and nucleotide were measured by HPLC system (1260 Infinity, Diode Array Detector, Agilent). Volatile compounds were detected by triple quadrupole GC–MS (8890-7000E, Agilent). Volatile aroma compounds were determined by electronic nose (AIRSENSE, Beijing Yingsheng Hengtai Technology Co., Ltd.),

### 2.3 Determination of volatile and non-volatile nutritional components

#### 2.3.1 Determination of crude polysaccharide content

Crude polysaccharides were determined by the phenol–sulfuric acid method (Ren, 2025). Ground BC and CS powders (2.000 ± 0.005 g each; *n* = 3) were weighed into 50 mL centrifuge tubes, mixed with 20 mL of 80% ethanol, and ultrasonically extracted for 30 min. After centrifugation (8000 r/min, 10 min), the supernatant was discarded, and the residue was washed with 80% ethanol and centrifuged again. The residue was then extracted twice with 30 mL of ultrapure water under ultrasonication for 30 min each. The combined filtrate was made up to 100 mL with ultrapure water and diluted for analysis. A standard glucose solution (0.1 g/L) was prepared, and aliquots (0, 0.2, 0.4, 0.6, 0.8, and 1.0 mL) were made up to 1 mL with ultrapure water, the absorbance was measured at 490 nm. The standard curve was plotted using glucose concentration as the abscissa and absorbance as the ordinate. For determination, 1 mL of sample or standard solution was mixed sequentially with 1 mL of 5% phenol and 5 mL of concentrated sulfuric acid. After standing for 10 min, the mixture was vortexed and incubated in a 30 °C water bath for 20 min, and the absorbance was measured at 490 nm. Polysaccharide contents in BC and CS were calculated from the standard curve.

#### 2.3.2 Determination of soluble protein content

Soluble proteins were determined by the Coomassie brilliant blue method (Santos et al., 2023). BC and CS powders (2.000 ± 0.005 g each; *n* = 3) were ground with ultrapure water, and the homogenate was transferred into a 50 mL centrifuge tube and ultrasonicated for 1 h. After centrifugation (8000 r/min, 10 min), the supernatant was made up to 100 mL with ultrapure water and diluted for analysis. A bovine serum albumin (BSA) solution (100 μg/mL) was prepared, and aliquots (0, 0.2, 0.4, 0.6, 0.8, and 1.0 mL) were made up to 1 mL with ultrapure water, the absorbance was measured at 595 nm. The standard curve was plotted using BSA concentration as the abscissa and absorbance as the ordinate. For determination, 1 mL of sample or standard solution was mixed with 5 mL of Coomassie brilliant blue G-250, and the absorbance was measured at 595 nm after 2 min. Soluble protein contents in BC and CS were calculated from the standard curve.

#### 2.3.3 Determination of free amino acid content

Free amino acids were determined by a modified literature method (Che, 2013). BC and CS powders (2.000 ± 0.005 g each; *n* = 3) were weighed into 10 mL centrifuge tubes, mixed with 5 mL of boric acid buffer (pH 9.0), and kept at 4 °C overnight. After ultrasonication for 1 h and centrifugation (10,000 r/min, 15 min), the supernatant was made up to 10 mL with boric acid buffer. Standard solutions of 20 amino acids (1 mg/mL each) were prepared, and 50 μL of each was mixed and diluted with boric acid buffer to obtain a series of concentrations. Standard solutions were analyzed by HPLC. The standard curve was plotted for each amino acid using its concentration as the abscissa and peak area as the ordinate. For determination, 600 μL of CNBF solution was added to the sample or mixed standard solution, and the volume was made up to 2 mL with boric acid buffer. After mixing, the mixture was derivatized in a 60 °C water bath for 30 min, filtered through a 0.22 μm membrane, and analyzed by HPLC. Free amino acid contents in BC and CS were calculated from the standard curves.

Chromatographic conditions: Agilent 1260 with a DAD detector; Welch Ultimate® Amino Acid column (4.6 × 250 mm, 5 μm); mobile phase A, sodium acetate–triethylamine solution (pH 6.20); mobile phase B, 64% acetonitrile; flow rate, 0.8 mL/min; injection volume, 10 μL; column temperature, 40 °C; detection wavelength, 254 nm. Elution program: 0–11 min, B 35 → 46%; 11–30 min, B 46 → 80%; 30–35 min, B 80 → 100%; 35–40 min, B 100%; 40–42 min, B 100 → 35%; 42–47 min, B 35%. Mobile phases A and B were combined to 100% at each time point.

#### 2.3.4 Determination of nucleotide content

Nucleotides were determined by a modified literature method (Dong et al., 2018). BC and CS powders (2.000 ± 0.005 g each; *n* = 3) were mixed with 5 mL of ultrapure water, boiled at 100 °C for 10 min, and centrifuged (10,000 r/min, 10 min) at room temperature. The filtrate was made up to 10 mL with ultrapure water. Standard solutions of six 5′-nucleotides (1 mg/mL each) were prepared, and 100 μL of each was mixed and diluted with ultrapure water to obtain a series of concentrations. Standard solutions were analyzed by HPLC. The standard curve was plotted for each 5′-nucleotide using its concentration as the abscissa and peak area as the ordinate. The sample and mixed standard solutions were filtered through a 0.22 μm membrane and analyzed by HPLC. 5′-Nucleotide contents in BC and CS were calculated from the standard curves.

Chromatographic conditions: Agilent 1260 with a DAD detector; Eclipse XDB-C18 column (4.6 × 250 mm, 5 μm); mobile phase A, methanol; mobile phase B, 10 mM KH₂PO₄; flow rate, 1 mL/min; injection volume, 10 μL; column temperature, 30 °C; detection wavelength, 254 nm. Elution program: 0–3 min, B 95 → 96%; 3–8 min, B 96 → 97%; 8–15 min, B 97 → 100%; 15–18 min, B 100 → 95%; 18–23 min, B 95%. Mobile phases A and B were combined to 100% at each time point.

#### 2.3.5 Determination of crude fiber content

Crude fiber was determined according to GB/T 5009.10–2003 (Institute of Health Supervision and Inspection of Food, M. o. H., 2003). Filter paper and a glass dish were dried in a constant-temperature oven, cooled, and weighed. BC and CS powders (5.000 ± 0.005 g each; *n* = 3) were placed into 250 mL conical flasks, and 200 mL of boiling 1.25% H₂SO₄ was added. After heating for 30 min, the mixture was filtered under suction, and the residue was washed with boiling water until no longer acidic and returned to the flask. Then, 200 mL of boiling 1.25% KOH was added, and the mixture was heated for 30 min and filtered under suction. The residue was washed with boiling water until no longer alkaline, rinsed with anhydrous ethanol, transferred with the filter paper into the glass dish, dried thoroughly, cooled, and weighed.

#### 2.3.6 Determination of volatile compounds

Volatile compounds were determined using the headspace method (Yin et al., 2019). BC and CS powders (5.00 g each; *n* = 3) were placed into 20 mL headspace vials and heated at 90 °C for 30 min. Chromatographic conditions: SH-I-5Sil MS column (30 m × 250 μm × 0.25 μm); flow rate, 1.0 mL/min; temperature program: 40 °C held for 2 min, increased to 180 °C at 3 °C/min and held for 2 min, then increased to 220 °C at 10 °C/min and held for 1 min; inlet temperature, 220 °C; split ratio, 10:1; carrier gas, helium. Mass spectrometric conditions: electron ionization at 70 eV; mass scan range, 50–500 *m*/*z*; transfer line temperature, 280 °C; ion source temperature, 230 °C. Volatile compounds (alcohols, ketones, esters, aldehydes, etc.) in BC and CS were identified against the NIST 20.L library, and their relative contents were calculated by the normalization method.

### 2.4 Texture determination

Texture Profile Analysis (TPA) was performed on the pileus and stipe of BC and CS to measure hardness, springiness, cohesiveness, gumminess, and chewiness. TPA parameters: 75 mm flat disk probe; pre-test, test, and pos*t*-test speeds of 60, 60, and 120 mm/min, respectively; 60% deformation; 2 s interval; and 0.1 N trigger force. All measurements were performed in triplicate (*n* = 3 biological replicates).

### 2.5 Equivalent umami concentration (EUC) calculation

Equivalent Umami Concentration (EUC) quantifies umami intensity as the total umami-active substance content in 100 g of material, expressed in monosodium glutamate (MSG) equivalents. It was calculated according to Wen X et al. (Wen et al., 2022) using the following formula:

$$Y=\sum a_{i}b_{i}+1218\left(\sum a_{i}b_{i}\right)\left(\sum a_{j}b_{j}\right)$$

**Table:** 

Y	–	Equivalent Umami Concentration (g MSG/100 g)；
a_i_	–	concentration of umami amino acids in the sample (g/100 g)；
a_j_	–	concentration of umami nucleotides in the sample (g/100 g)；
b_i_	–	umami coefficient of umami amino acids relative to MSG (Glu = 1.000, Asp = 0.077)；
b_j_	–	umami coefficient of umami nucleotides relative to 5’-IMP (5’-IMP = 1.00, 5’-GMP = 2.30, 5’-XMP = 0.61, 5’-AMP = 0.18)；
1218	–	synergistic effect constant.

### 2.6 Calculation of taste activity value (TAV)

Taste Activity Value (TAV) indicates the contribution of a substance to sample flavor, with TAV > 1 denoting a significant contribution and TAV < 1 a minor one (Zhao, 2022). Taste characteristics and classification of free amino acids (Wang et al., 2020), along with calculation methods and taste thresholds for flavor-active substances, were referenced from the literature (Wei, 2020; Zhao, 2022). The calculation formula is as follows:

$$\mathrm{TAV}=\frac{\text{concentration of the taste}-\text{active substance}}{\text{taste threshold of the taste}-\text{active substance}}$$

### 2.7 Calculation of relative odor activity value (ROAV)

Relative Odor Activity Value (ROAV) indicates the contribution of a substance to sample aroma: ROAV ≥1 denotes a significant contribution, and 0.1 ≤ ROAV <1 a modifying role (Zhang, 2023). Aroma descriptors were obtained from The Good Scents Company database (https://www.thegoodscentscompany.com/), and aroma thresholds in water from *Compendium of Odor Threshold Values for Chemical Compounds* (2nd ed.) (Van, 2018). OAV is the ratio of substance concentration to aroma threshold, calculated as previously described (Liu et al., 2008):

$$\text{ROAV}=\frac{\mathrm{OAV}}{{\mathrm{OAV}}_{\max}}\times 100$$

### 2.8 Electronic tongue analysis

BC and CS powders (∼5.00 g each, *n* = 3) were boiled in 75 mL ultrapure water for 10 min, centrifuged at 8000 r/min for 15 min at room temperature, and the supernatants were collected for analysis.

### 2.9 *Re*-addition experiment of taste-active substances

To validate the correlation results, free amino acids (*P* < 0.01) and 5′-nucleotides (*P* < 0.05) were spiked into BC and CS extracts at double their original concentrations, followed by electronic tongue analysis.

### 2.10 Electronic nose analysis

BC and CS powders (∼5.00 g each, *n* = 3) were placed in 20 mL sampling vials and heated at 90 °C for 30 min. Acquisition parameters: sampling interval, 1 s; self-cleaning, 80 s; sampling time, 5 s; injection flow rate, 400 mL/min. Original sensor response curves were recorded.

### 2.11 In vitro anticancer activity assay

#### 2.11.1 Cell culture

Human colon cancer cells (HCT116) and human gastric cancer cells (HGC27) were selected as model cell lines. Cells at 80%–90% confluence were harvested. The original culture medium was discarded, and the cells were rinsed 1–2 times with sterile enzyme-free PBS buffer. For each 25 cm^2^ culture flask, 1 mL of trypsin pre-warmed to 37 °C was added, and the cells were incubated at 37 °C in a 5% CO₂ incubator. After the cells became rounded and detached from the flask surface, complete medium (at a volume five times that of the trypsin) was added to terminate the digestion. The cell suspension was then centrifuged at 100 ×*g* for 5 min, after which the supernatant was discarded. An appropriate volume of complete medium was added, and the cells were gently resuspended by pipetting. Subsequently, cell counting was performed using trypan blue staining solution.

#### 2.11.2 Cytotoxicityassay

Cells were seeded into 96-well plates at densities of 2.5 × 10^3^ cells/100 μL (HGC27) and 7 × 10^3^ cells/100 μL (HCT116) and incubated at 37 °C/5% CO₂ for 24 h. The medium was then replaced with drug-containing RPMI 1640 (HGC27) or DMEM (HCT116), and cells were incubated for an additional 48 h; controls received drug-free medium. After removing the supernatant, 110 μL of CCK-8 solution (10% in incomplete medium) was added to each well, followed by incubation at 37 °C in the dark for 1 h. Absorbance was measured at 450 nm, and cell viability was calculated as follows:

$$\text{cell viability}=\frac{\text{absorbance}\;\mathrm{per}\;\text{well}}{\text{average absorbance of the control}}\times 100\%$$

#### 2.11.3 Determination of cellular reactive oxygen species (ROS)

Cellular ROS levels were measured using a slightly modified protocol (Song et al., 2021). HGC27 and HCT116 cells were seeded into 6-well plates at densities of 2 × 10^5^ and 6 × 10^5^ cells/2 mL, respectively, and incubated at 37 °C/5% CO₂ for 24 h. The medium was then replaced with drug-containing RPMI 1640 (HGC27) or DMEM (HCT116) for 3 h; controls received drug-free medium. After washing with PBS, cells were detached with 250 μL pre-warmed EDTA-free trypsin (37 °C) and quenched with 1 mL complete medium. Following centrifugation at 300 ×*g* for 5 min, cells were washed twice with PBS (300 ×g, 5 min each) and resuspended in 1 mL DCFH-DA solution (1:1500 in incomplete medium). After incubation at 37 °C in the dark for 30 min, cells were washed twice with PBS and resuspended in 500 μL PBS for flow cytometric analysis.

### 2.12 Data processing

The experimental data were preliminarily organized and processed using Excel 2019. Significance analysis was performed using IBM SPSS Statistics 27 software. The data are expressed as mean ± standard deviation (SD). All figures were plotted using Origin 2021, RStudio, and GraphPad Prism 10.1.2. Significance analysis and IC₅₀ fitting were performed using GraphPad Prism 10.1.2. Statistical analysis was performed using SPSS with the t-test.

## 3 Results

### 3.1 Contents of crude polysaccharides, soluble protein, and crude fiber

As shown in Fig. 1, the contents of crude polysaccharides, soluble protein, and crude fiber in BC were all significantly higher than those in CS. The contents in BC were 0.5038 ± 0.0064 g/100 g, 4.0894 ± 0.0660 mg/g, and 2.6001 ± 0.2395%, respectively, whereas the contents in CS were 0.3984 ± 0.0306 g/100 g, 3.5188 ± 0.1135 mg/g, and 1.7724 ± 0.1261%, respectively.

**Fig. 1:**
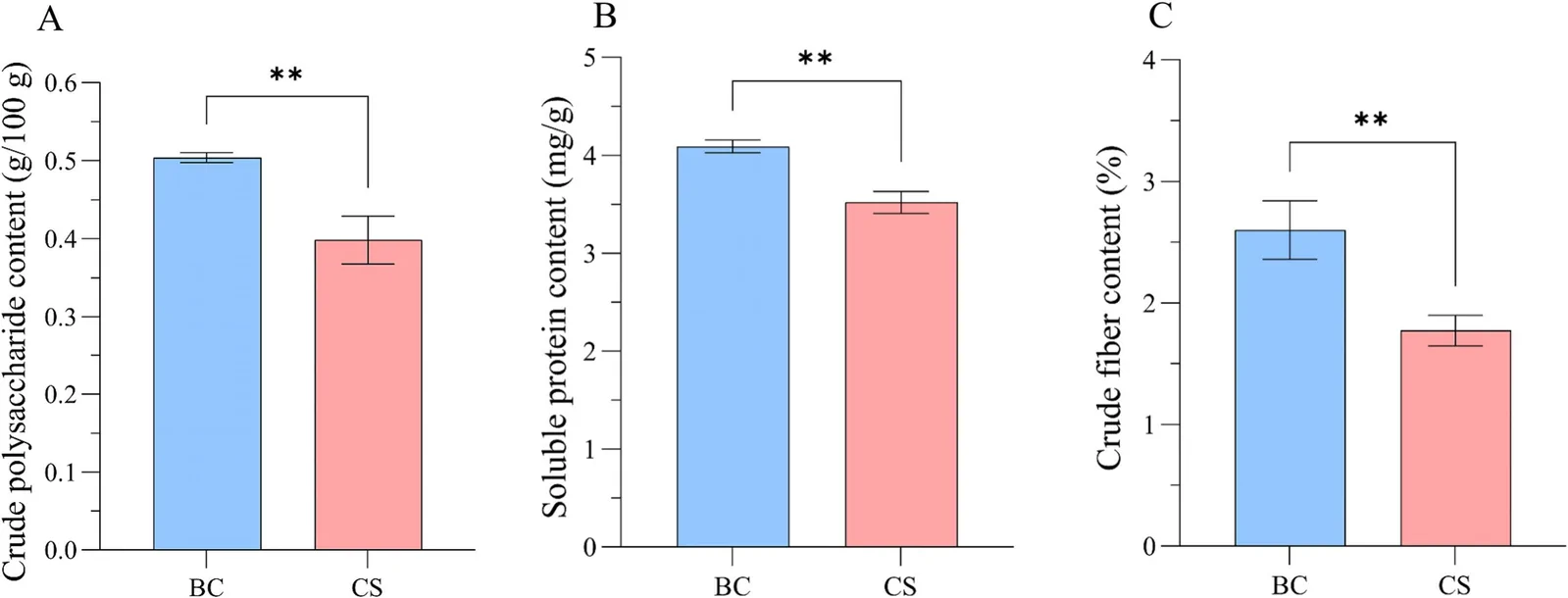
(A) Crude polysaccharide content; (B) Soluble protein content; (C) Crude fiber content. Asterisks indicate significant differences between groups (** *P* < 0.01).

### 3.2 Texture characteristics

Texture characteristics are critical indicators of edible mushroom quality. Hardness measures resistance to compressive deformation; cohesiveness reflects internal structural integrity; springiness indicates elastic recovery after stress removal; gumminess represents resistance to chewing deformation; and chewiness integrates hardness, springiness, and cohesiveness, reflecting the energy required to reach a swallowable state. TPA results (Table 1) revealed comparable cohesiveness between BC and CS in both pileus and stipe. However, BC exhibited significantly higher hardness, gumminess, and chewiness than CS across both tissues. While pileus springiness did not differ significantly between strains, BC stipe springiness was significantly higher than that of CS.

**Table 1:** Texture characteristic of BC and CS.

Sample part		Hardness (N)	Cohesiveness (Ratio)	Resilience (mm)	Adhesiveness (mj)	Chewiness (mj)
Cap	BC	5.96 ± 0.60^a^	0.35 ± 0.04^a^	1.94 ± 0.26^a^	2.09 ± 0.15^a^	4.06 ± 0.69^a^
	CS	3.99 ± 0.64^b^	0.32 ± 0.04^a^	1.61 ± 0.26^a^	1.28 ± 0.23^b^	2.10 ± 0.70^b^
Stem	BC	40.50 ± 3.04^a^	0.32 ± 0.04^a^	2.15 ± 0.42^a^	12.97 ± 2.24^a^	28.48 ± 10.00^a^
	CS	33.92 ± 1.40^b^	0.25 ± 0.02^a^	1.87 ± 0.24^b^	7.77 ± 0.92^b^	14.62 ± 3.11^b^

Note: Different lowercases indicate significant differences between groups (*P* < 0.05).

### 3.3 Electronic tongue analysis

The electronic tongue quantifies basic tastes (sourness, bitterness, saltiness, sweetness, and umami) by mimicking human gustation. As shown in Fig. 2A, BC and CS showed comparable saltiness, whereas BC exhibited higher sourness, bitterness, sweetness, and umami, suggesting a richer flavor profile. OPLS-DA (Fig. 2B) showed that Components 1 and 2 explained 70.3% and 17.1% of the variance, respectively (cumulative 87.4%), with clear intra-group clustering and inter-group separation, indicating distinct taste profiles between the two strains. Model validation (Fig. 2C) yielded Q^2^ = 0.942 and R^2^Y = 0.971, demonstrating robust reliability and predictive power.

**Fig. 2:**
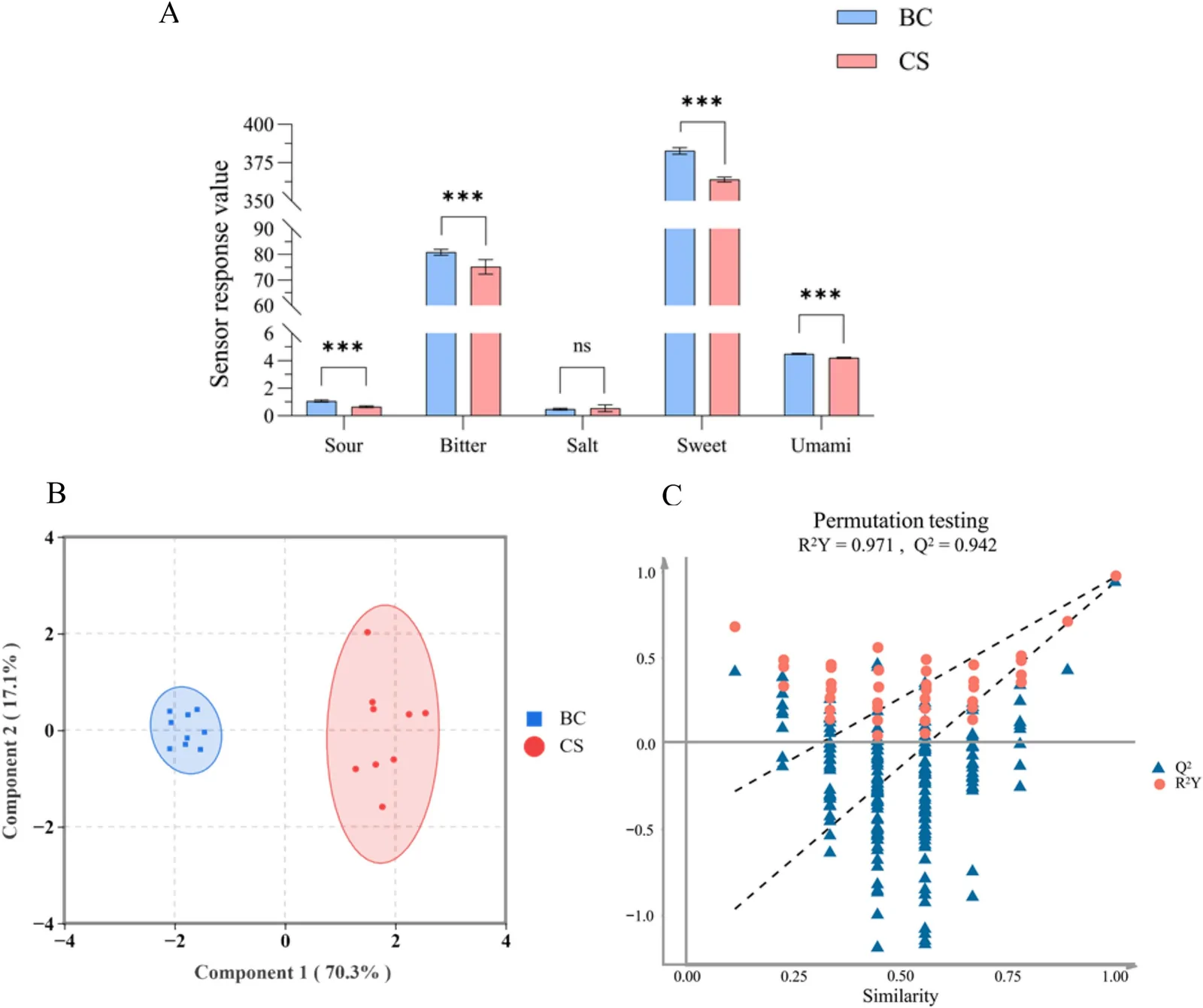
(A) Response values of electronic tongue sensors; (B) OPLS-DA score plot; (C) OPLS-DA validation plot. Asterisks indicate significant differences between groups (*** *P* < 0.001).

### 3.4 Determination of free amino acid content

Free amino acids are central to edible mushroom flavor. In this study, 20 free amino acids were analyzed (standard chromatogram was shown in Fig. 3), of which 18 were detected in both BC and CS (chromatograms were shown in Fig. S1); cysteine and isoleucine were absent. Total free amino acid contents were 15.18 ± 0.18 mg/g in BC and 10.78 ± 0.19 mg/g in CS. While the amino acid profiles were identical between the two strains, each individual component was significantly more abundant in BC. Based on taste characteristics, amino acids were classified into four categories: umami (Asp, Glu, Lys), bitter (Val, Met, Arg, Trp, Leu), aromatic (Phe, Tyr), and sweet (Gly, Pro, Ala, Thr, Ser, His). Category totals and individual TAV values are presented in Table 2, Table 3. TAV analysis identified seven taste-active amino acids in BC (aspartic acid, glutamic acid, lysine, arginine, valine, alanine, and histidine) versus five in CS (glutamic acid, lysine, arginine, valine, and alanine). BC exhibited significantly higher total contents of umami, bitter, aromatic, and sweet amino acids than CS.

**Fig. 3:**
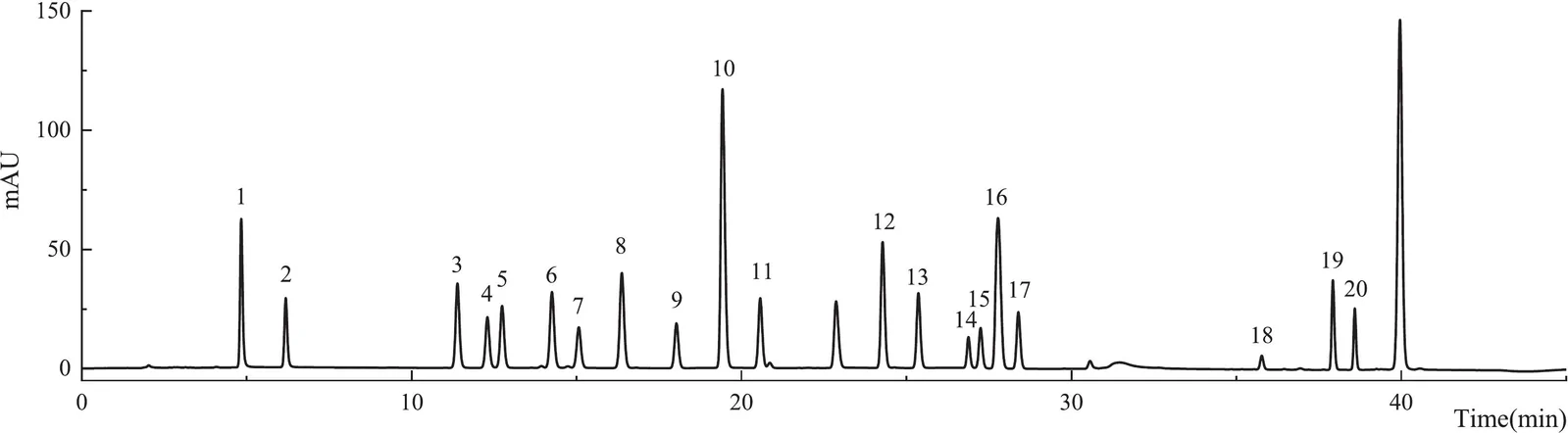
HPLC (Diode Array Detector) chromatogram of amino acid mixed standards. Note: 1. Aspartic acid (Asp); 2. Glutamic acid (Glu); 3. Asparagine (Asn); 4. Histidine (His); 5. Glutamine (Gln); 6. Serine (Ser); 7. Arginine (Arg); 8. Threonine (Thr); 9. Proline (Pro); 10. Glycine (Gly); 11. Alanine (Ala); 12. Valine (Val); 13. Methionine (Met); 14. Tryptophan (Trp); 15. Leucine (Leu); 16. Isoleucine (Ile); 17. Phenylalanine (Phe); 18. Cysteine (Cys); 19. Tyrosine (Tyr); 20. Lysine (Lys).

**Table 2:** Amino acid composition and their contents.

Taste characteristics	Free amino acid	Free amino acid content (mg/g)		Threshold (mg/g)	TAV	
		BC	CS		BC	CS
Umami	Asp	1.43 ± 0.10^a^	0.87 ± 0.00^b^	1.00	1.43 ± 0.10^a^	0.87 ± 0.00^b^
Umami	Glu	1.36 ± 0.01^a^	1.04 ± 0.03^b^	0.30	4.53 ± 0.03^a^	3.48 ± 0.08^b^
Umami	Lys	1.18 ± 0.01^a^	0.71 ± 0.03^b^	0.50	2.37 ± 0.02^a^	1.42 ± 0.06^b^
Bitter	Arg	0.95 ± 0.02^a^	0.57 ± 0.01^b^	0.50	1.91 ± 0.03^a^	1.14 ± 0.02^b^
Bitter	Val	0.58 ± 0.00^a^	0.43 ± 0.00^b^	0.40	1.45 ± 0.01^a^	1.07 ± 0.01^b^
Bitter	Met	0.13 ± 0.00^a^	0.07 ± 0.00^b^	0.30	0.42 ± 0.01^a^	0.24 ± 0.01^b^
Bitter	Trp	0.25 ± 0.00^a^	0.21 ± 0.00^b^	0.90	0.28 ± 0.00^a^	0.24 ± 0.00^b^
Bitter	Leu	0.71 ± 0.00^a^	0.55 ± 0.01^b^	1.90	0.38 ± 0.00^a^	0.29 ± 0.00^b^
Aroma	Phe	0.35 ± 0.00^a^	0.29 ± 0.01^b^	0.90	0.39 ± 0.00^a^	0.32 ± 0.01^b^
Aroma	Tyr	2.52 ± 0.05^a^	2.37 ± 0.07^b^	2.60	0.97 ± 0.02^a^	0.91 ± 0.03^b^
Sweet	Gly	0.41 ± 0.00^a^	0.27 ± 0.01^b^	1.30	0.31 ± 0.00^a^	0.21 ± 0.01^b^
Sweet	Ala	1.13 ± 0.02^a^	0.78 ± 0.02^b^	0.60	1.88 ± 0.03^a^	1.29 ± 0.04^b^
Sweet	Thr	0.67 ± 0.01^a^	0.47 ± 0.00^b^	2.60	0.26 ± 0.00^a^	0.18 ± 0.00^b^
Sweet	Pro	0.36 ± 0.00^a^	0.28 ± 0.01^b^	3.00	0.12 ± 0.00^a^	0.09 ± 0.00^b^
Sweet	His	0.26 ± 0.00^a^	0.19 ± 0.00^b^	0.20	1.31 ± 0.02^a^	0.95 ± 0.01^b^
Sweet	Ser	0.78 ± 0.01^a^	0.56 ± 0.01^b^	1.50	0.52 ± 0.01^a^	0.37 ± 0.01^b^
/	Gln	1.63 ± 0.02^a^	0.77 ± 0.02^b^	/	/	/
/	Asn	0.48 ± 0.01^a^	0.35 ± 0.01^b^	/	/	/

Note: Different lowercases indicate significant differences between groups (*P* < 0.05), “/” indicates no result found.

**Table 3:** Taste-active amino acids content.

Free amino acids	Content (mg/g)	
	BC	CS
Total umami amino acids	3.97 ± 0.11^a^	2.63 ± 0.04^b^
Total bitter amino acids	2.63 ± 0.04^a^	1.83 ± 0.03^b^
Total aroma amino acids	2.87 ± 0.05^a^	2.66 ± 0.07^b^
Total sweet amino acids	3.60 ± 0.03^a^	2.54 ± 0.05^b^
Total amino acids	15.18 ± 0.18^a^	10.78 ± 0.19^b^

Note: Different lowercases indicate significant differences between groups (*P* < 0.05).

### 3.5 Determination of 5′-nucleotide content

5′-Nucleotides synergize with taste-active amino acids to modulate mushroom flavor. Six 5′-nucleotides were analyzed (mixed standard chromatogram was shown in Fig. 4A), of which five were detected in both BC and CS (chromatograms were shown in Fig. S2); 5′-inosinic acid (5′-IMP) was absent (Fig. 4B). Total 5′-nucleotide content was significantly higher in BC (1.39 ± 0.04 mg/g) than in CS (0.93 ± 0.05 mg/g). Although the nucleotide profiles were identical, individual components differed: 5′-adenylic acid (5′-AMP) showed comparable levels, whereas 5′-cytidylic acid (5′-CMP) was more abundant in BC, and 5′-uridylic acid (5′-UMP), 5′-guanylic acid (5′-GMP), and 5′-xanthylic acid (5′-XMP) were more abundant in CS.

**Fig. 4:**
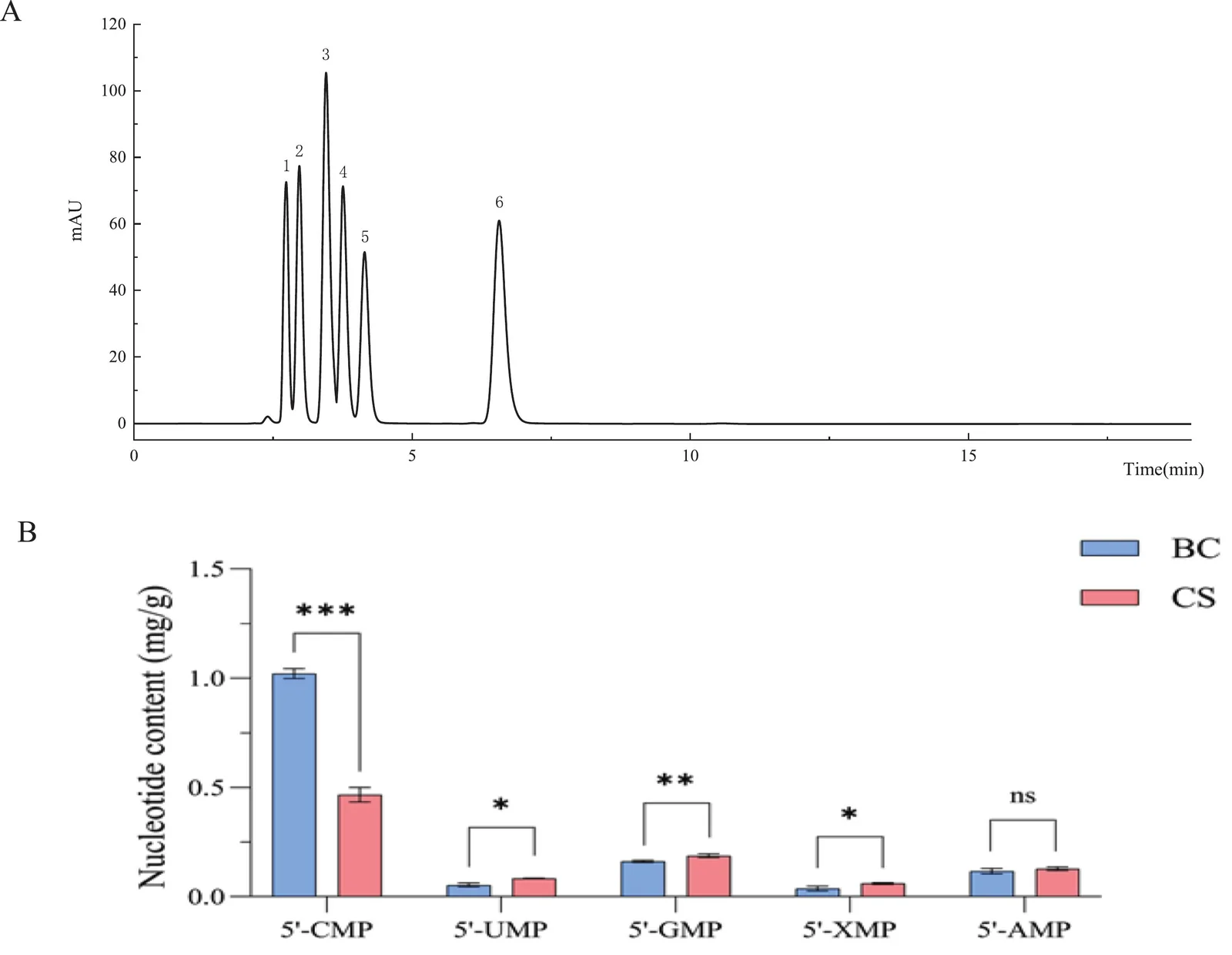
(A) HPLC (Diode Array Detector) chromatogram of 5′-nucleotides mixed standards; (B) 5′-nucleotide content. Asterisks indicate significant differences between groups (*** *P* < 0.001, ** *P* < 0.01, * *P* < 0.05). Note: 1. 5’-CMP; 2. 5’-UMP; 3. 5’-GMP; 4. 5’-IMP; 5. 5’-XMP; 6. 5’-AMP.

### 3.6 Calculation of equivalent umami concentration

The calculation of equivalent umami concentration (EUC) values was performed by applying the formula to the umami amino acids and umami nucleotides in BC and CS. As shown in Fig. 5, the EUC value of BC was 7.5681 ± 0.1833 g MSG/100 g, which was significantly higher than that of CS.

**Fig. 5:**
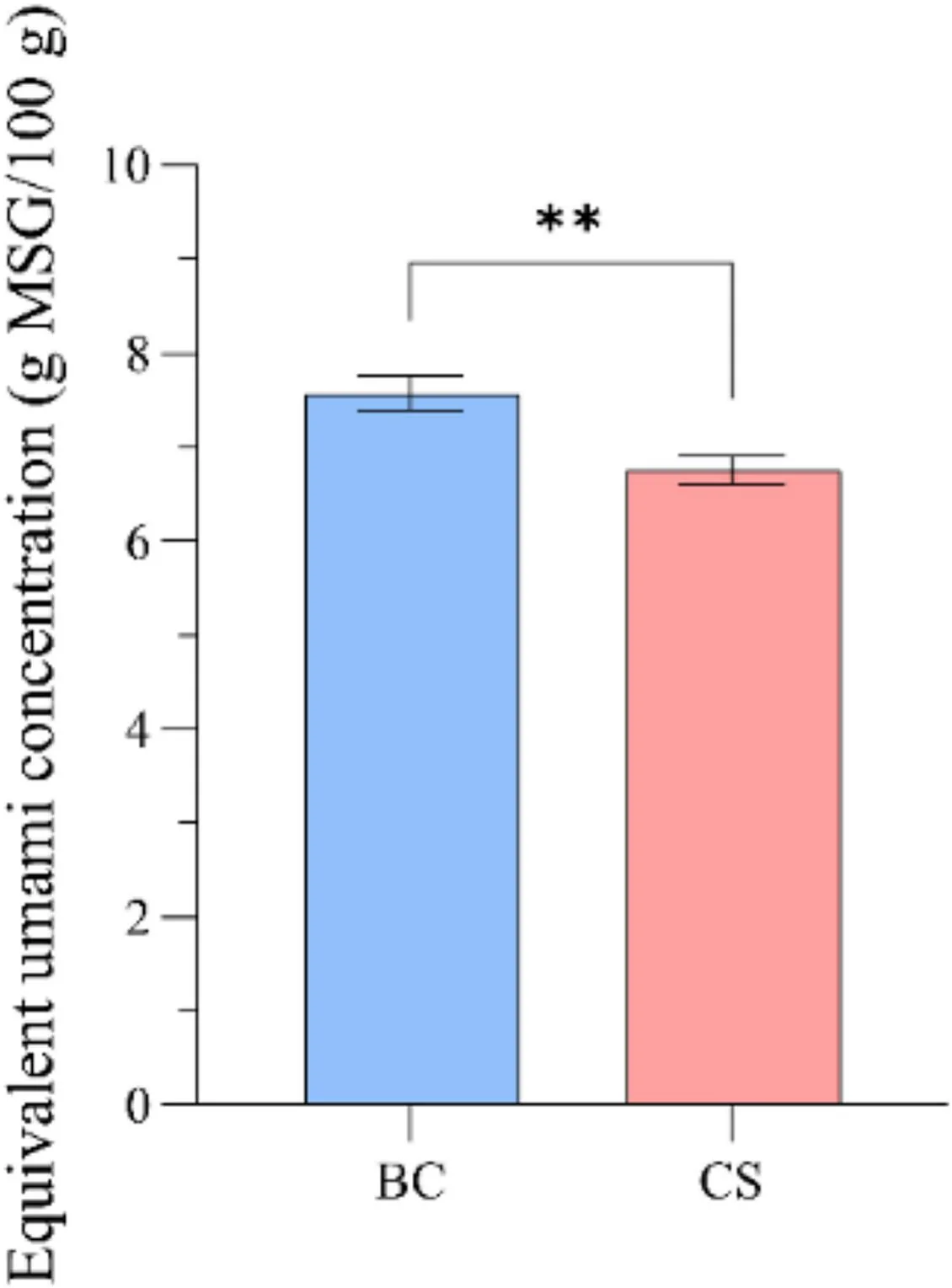
Comparison chart of EUC. Asterisks indicate significant differences between groups (** *P* < 0.01).

### 3.7 Correlation analysis of taste compounds and electronic tongue

To identify key taste compounds driving the flavor profiles of BC and CS, correlation analysis was performed between free amino acids, 5′-nucleotides, and electronic tongue sensor values (Fig. 6). Sourness correlated positively with aspartic acid, glutamine, arginine, valine, tryptophan, tyrosine, lysine, and 5′-cytidylic acid; bitterness with glutamic acid, aspartic acid, serine, arginine, threonine, proline, glycine, alanine, valine, phenylalanine, and tyrosine; sweetness with asparagine, histidine, glycine, alanine, methionine, tryptophan, tyrosine, and lysine; and umami with aspartic acid, glutamine, tryptophan, and lysine. To validate these correlations, significantly correlated free amino acids (*P* < 0.01) and 5′-nucleotides (*P* < 0.05) were spiked into BC and CS extracts (Table 4). The addition-back experiments confirmed that aspartic acid, glutamine, tryptophan, lysine, and 5′-cytidylic acid contributed to sourness, while glutamine and tryptophan contributed to umami.

**Fig. 6:**
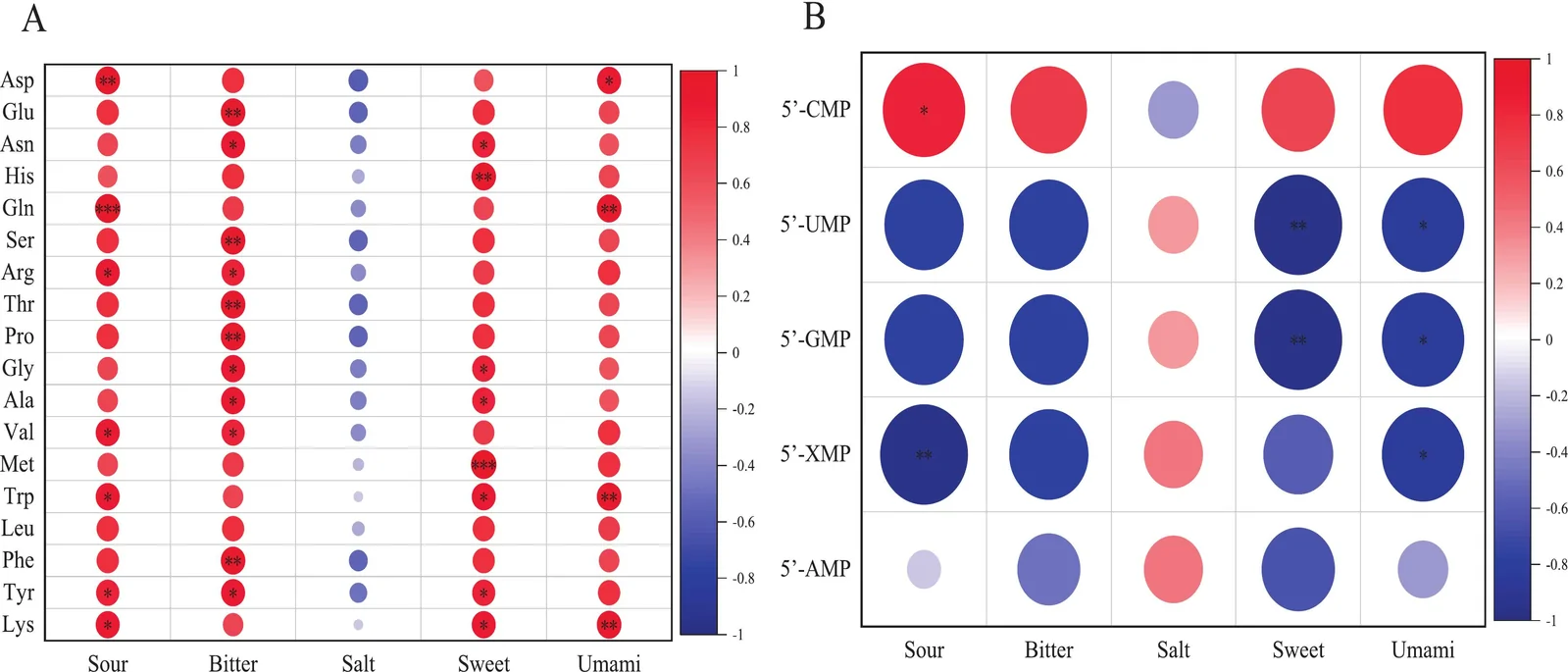
(A) Correlation analysis between amino acids and electronic tongue taste; (B) Correlation analysis between 5′-nucleotides and electronic tongue taste. Asterisks indicate significant correlation between groups (*** *P* < 0.001, ** *P* < 0.01, * *P* < 0.05).

**Table 4:** Electronic tongue taste re-addition test results.

Flavor compounds	Sour	Bitter	Sweet	Umami
Asp	+	/	−	−
Glu	+	/	−	−
His	+	−	/	/
Gln	+	−	−	+
Ser	+	−	−	/
Thr	+	−	−	+
Pro	/	/	−	+
Met	/	+	−	+
Trp	+	−	−	+
Phe	+	/	−	+
Lys	+	−	−	/
5’-CMP	+	−	−	+
Mix	+	−	−	+

Note: “+” indicates the sensor values of the electronic tongue increase after adding the substance; “-” indicates the sensor values of the electronic tongue reduce after adding the substance; “/” indicates no change.

### 3.8 Electronic nose measurement

The electronic nose mimics human olfaction to quantify sample odor profiles. As shown in Fig. 7A, W5S, W1W, and W2W sensors exhibited relatively high responses, with BC exceeding CS. These sensors detect nitrogen oxides, sulfides, aromatic hydrocarbons, and sulfur-containing organics, respectively. OPLS-DA (Fig. 7B) showed that Components 1 and 2 explained 51.8% and 22.8% of the variance, respectively (cumulative 74.6%), with clear intra-group clustering and inter-group separation indicating distinct volatile profiles between strains. Model validation (Fig. 7C) yielded Q^2^ = 0.914 and R^2^Y = 0.972, confirming robust reliability.

**Fig. 7:**
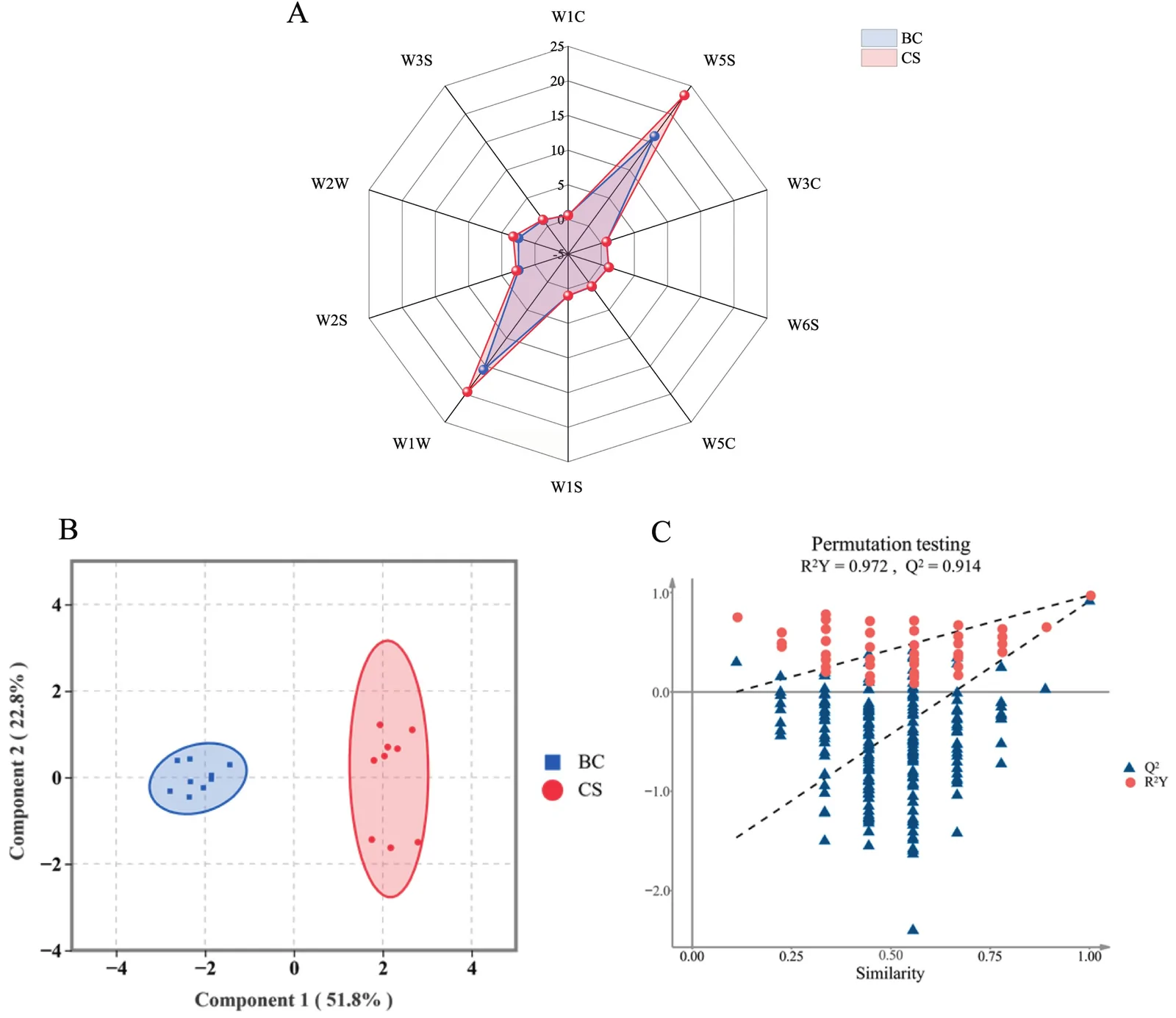
(A) Electronic nose radar chart; (B) OPLS-DA score plot; (C) OPLS-DA validation plot.

### 3.9 Volatile compounds

A total of 41 and 37 volatile compounds were identified in BC and CS, respectively, with 23 shared between them (Figs. 8A–B; Table 5, Table 6). Classified into ketones, esters, aldehydes, acids, alcohols, alkanes, and others, BC was dominated by aldehydes (13 compounds, 41.26%), ketones (8, 34.64%), and alkanes (8, 5.12%) in both diversity and content, whereas CS was dominated by ketones (8, 48.15%), aldehydes (11, 18.78%), and alcohols (8, 16.75%). BC contained higher proportions of aldehydes, acids, alkanes, and other compounds, while CS was richer in ketones, esters, and alcohols.

**Fig. 8:**
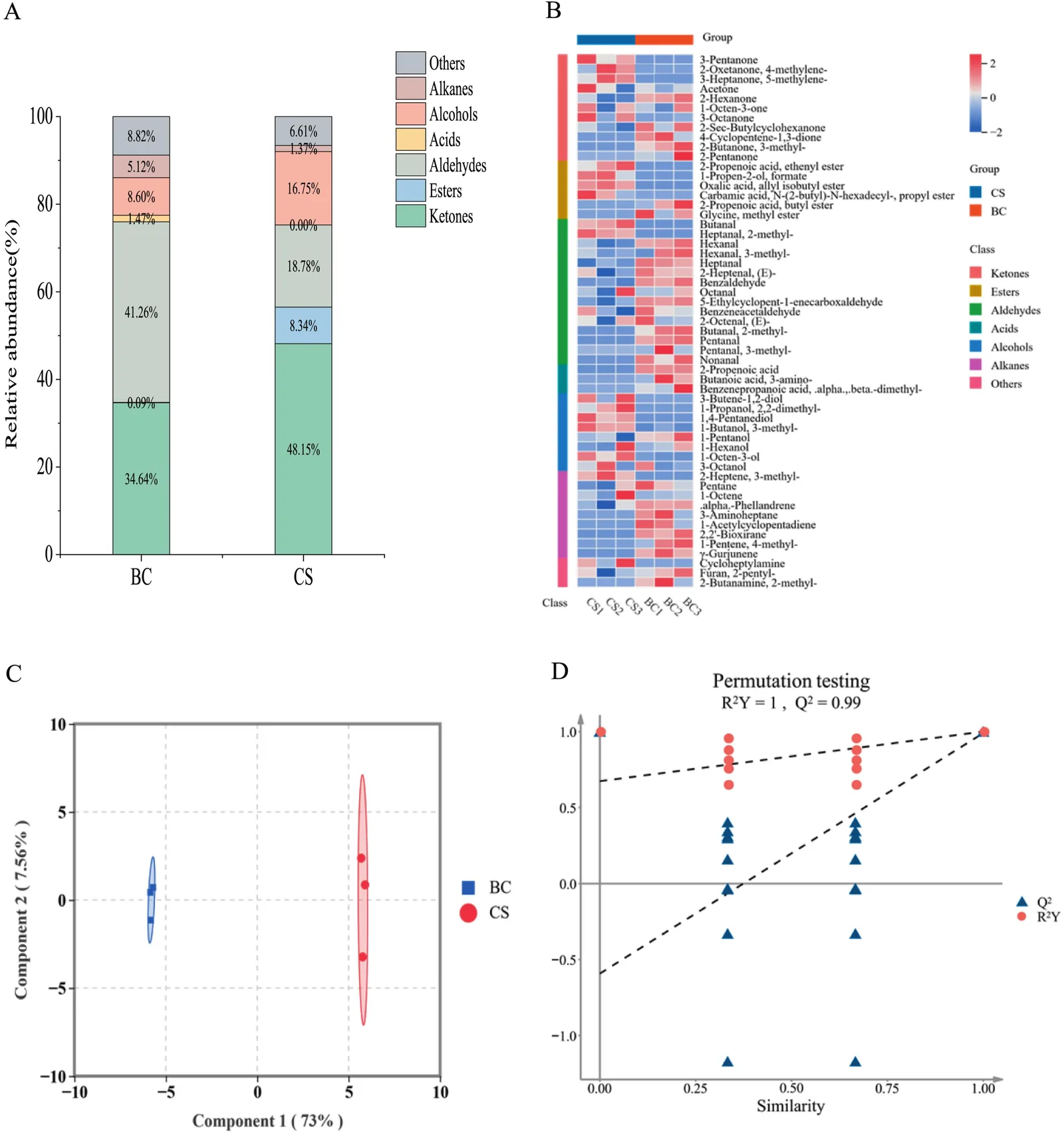
(A) Relative content of volatile component types; (B) Heatmap of relative content of volatile components; (C) OPLS-DA score plot; (D) OPLS-DA validation plot.

**Table 5:** The quantity of volatile compounds.

Compound categories	BC	CS
Ketones	8	8
Esters	2	4
Aldehydes	13	11
Acids	3	0
Alcohols	5	8
Alkanes	8	4
Others	2	2
Total	41	37

**Table 6:** GC–MS quantitative analysis.

Categories	Compounds	CAS	Relative content (%)	
			BC	CS
Ketones	3-Pentanone	96–22-0	ND	1.53%
	2-Oxetanone, 4-methylene-	674–82-8	ND	0.38%
	3-Heptanone, 5-methylene-	20,690–70-4	ND	0.01%
	Acetone	67–64-1	1.78%	2.69%
	2-Hexanone	591–78-6	1.26%	0.64%
	1-Octen-3-one	4312-99-6	0.43%	0.51%
	3-Octanone	106–68-3	29.84%	42.35%
	2-Sec-Butylcyclohexanone	14,765–30-1	0.06%	0.04%
	4-Cyclopentene-1,3-dione	930–60-9	0.03%	ND
	2-Butanone, 3-methyl-	563–80-4	0.12%	ND
	2-Pentanone	107–87-9	1.13%	ND
Esters	2-Propenoic acid, ethenyl ester	2177-18-6	ND	0.67%
	1-Propen-2-ol, formate	32,978–00-0	ND	4.28%
	Oxalic acid, allyl isobutyl ester	1,000,309–23-0	ND	3.36%
	Carbamic acid, N-(2-butyl)-N-hexadecyl-, propyl ester	1,000,491–06-9	ND	0.03%
	2-Propenoic acid, butyl ester	141–32-2	0.05%	ND
	Glycine, methyl ester	616–34-2	0.04%	ND
Aldehydes	Butanal	123–72-8	ND	0.65%
	Heptanal, 2-methyl-	16,630–91-4	ND	1.18%
	Hexanal	66–25-1	28.85%	15.12%
	Hexanal, 3-methyl-	19,269–28-4	0.06%	0.04%
	Heptanal	111–71-7	0.35%	0.10%
	2-Heptenal, (*E*)-	18,829–55-5	0.19%	0.16%
	Benzaldehyde	100–52-7	3.92%	0.79%
	Octanal	124–13-0	0.16%	0.19%
	5-Ethylcyclopent-1-enecarboxaldehyde	36,431–60-4	0.20%	0.10%
	Benzeneacetaldehyde	122–78-1	0.43%	0.39%
	2-Octenal, (E)-	2548-87-0	0.04%	0.05%
	Butanal, 2-methyl-	96–17-3	2.29%	ND
Aldehydes	Pentanal	110–62-3	2.77%	ND
	Pentanal, 3-methyl-	15,877–57-3	1.95%	ND
	Nonanal	124–19-6	0.04%	ND
Acids	2-Propenoic acid	79–10-7	1.39%	ND
	Butanoic acid, 3-amino-	541–48-0	0.01%	ND
	Benzenepropanoic acid, .alpha.,.beta.-dimethyl-	19,731–91-0	0.07%	ND
Alcohols	3-Butene-1,2-diol	497–06-3	ND	0.78%
	1-Propanol, 2,2-dimethyl-	75–84-3	ND	0.07%
	1,4-Pentanediol	626–95-9	ND	0.14%
	1-Butanol, 3-methyl-	123–51-3	0.30%	3.15%
	1-Pentanol	71–41-0	1.12%	0.88%
	1-Hexanol	111–27-3	2.50%	2.96%
	1-Octen-3-ol	3391-86-4	0.24%	3.06%
	3-Octanol	589–98-0	4.44%	5.72%
Alkanes	2-Heptene, 3-methyl-	3404-75-9	ND	0.14%
	Pentane	109–66-0	1.19%	1.05%
	1-Octene	111–66-0	0.10%	0.15%
	.alpha.-Phellandrene	99–83-2	0.03%	0.02%
	3-Aminoheptane	28,292–42-4	3.11%	ND
	1-Acetylcyclopentadiene	60,032–12-4	0.01%	ND
	2,2’-Bioxirane	1464-53-5	0.44%	ND
	1-Pentene, 4-methyl-	691–37-2	0.22%	ND
	γ-Gurjunene	22,567–17-5	0.01%	ND
Others	Cycloheptylamine	5452-35-7	ND	0.04%
	Furan, 2-pentyl-	3777-69-3	8.25%	6.57%
	2-Butanamine, 2-methyl-	594–39-8	0.57%	ND

ROAV analysis (Table 7) identified 20 aroma-active compounds across both strains. BC contained 12 significant contributors (ROAV ≥1) and 6 modifiers (0.1 ≤ ROAV <1), with 1-octen-3-one, hexanal, and 3-methylpentanal as the top contributors to its mushroom, herbal, and earthy notes. CS contained 8 significant contributors and 8 modifiers, with 1-octen-3-one, 1-octen-3-ol, and 3-octanone contributing to its mushroom, herbal, and fresh notes. The variations in the species and quantities of the key contributing compounds are responsible for the differences in their overall aroma.

**Table 7:** Aroma contribution of volatile compounds.

Compounds	CAS	Description of the aroma	Relative content (%)		Threshold (mg/kg)	ROAV	
			BC	CS		BC	CS
1-Octen-3-one	4312-99-6	Mushroom; earthy; musty	0.43	0.51	0.00005	100.00	100.00
Hexanal	66–25-1	Fresh; green; fatty	28.85	15.12	0.005	67.09	29.65
Pentanal, 3-methyl-	15,877–57-3	–	1.95	ND	0.00041	55.30	ND
Butanal, 2-methyl-	96–17-3	Musty; cocoa; phenolic	2.29	ND	0.001	26.63	ND
Furan, 2-pentyl-	3777-69-3	Fruity; green; earthy	8.25	6.57	0.0058	16.54	11.11
3-Octanone	106–68-3	Fresh; herbal; lavender	29.84	42.35	0.0214	16.21	19.40
1-Hexanol	111–27-3	Ethereal; fusel; oil	2.50	2.96	0.0056	5.19	5.18
Octanal	124–13-0	Aldehydic; waxy; citrus	0.16	0.19	0.000587	3.17	3.17
1-Octene	111–66-0	Gasoline	0.10	0.15	0.0005	2.33	2.94
Pentanal	110–62-3	Fermented; bready; fruity	2.77	ND	0.016	2.01	ND
1-Octen-3-ol	3391-86-4	Mushroom; earthy; green	0.24	3.06	0.0015	1.86	20.00
Heptanal	111–71-7	Fresh; aldehydic; fatty	0.35	0.10	0.0028	1.45	0.35
Benzeneacetaldehyde	122–78-1	Green; sweet; floral	0.43	0.39	0.0063	0.79	0.61
3-Octanol	589–98-0	Earthy; mushroom; herbal	4.44	5.72	0.078	0.66	0.72
Nonanal	124–19-6	Waxy; aldehydic; rose	0.04	ND	0.0011	0.42	ND
2-Hexanone	591–78-6	Fruity; fungal; meaty	1.26	0.64	0.04	0.37	0.16
Acetone	67–64-1	Solvent; ethereal; apple	1.78	2.69	0.08	0.26	0.33
2-Octenal, (*E*)-	2548-87-0	Fresh; cucumber; fatty	0.04	0.05	0.004	0.12	0.12
3-Pentanone	96–22-0	Ethereal; acetone	ND	1.53	0.04	ND	0.38
Butanal	123–72-8	Pungent; cocoa; musty	ND	0.65	0.009	ND	0.71

Note: ND indicates that the value was not detected, “—” indicates no result found.

OPLS-DA of GC–MS peak areas (Fig. 8C) showed that Components 1 and 2 explained 73% and 7.56% of the variance, respectively (cumulative 80.56%), with clear intra-group clustering and inter-group separation indicating distinct volatile profiles. Cross-validation (Fig. 8D) yielded Q^2^ > 0.5 (approaching 1) and R^2^Y = 1, confirming excellent model fit and stability.

### 3.10 In vitro anticancer activity

For HCT116 cells (Fig. 9A), BC extract (62.5–4000 μg/mL) reduced viability from 84.38% to 33.76% (IC50 = 1141 μg/mL), whereas CS extract (1000–12,000 μg/mL) reduced viability from 77.33% to 35.5% (IC50 = 5334 μg/mL). Both extracts inhibited HCT116 in a concentration-dependent manner, with BC markedly more potent than CS.

**Fig. 9:**
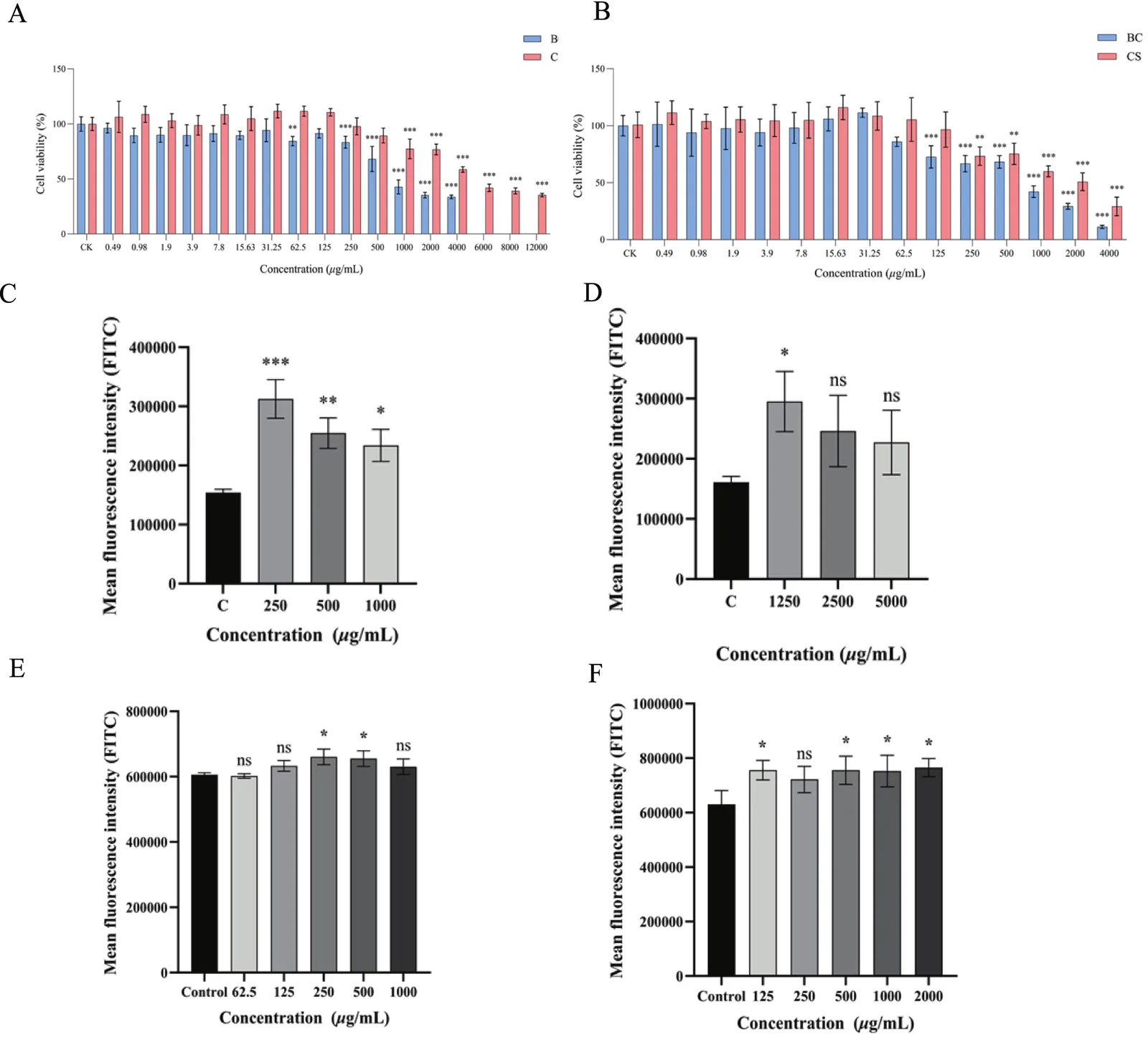
Anticancer activity of the alcohol extract. (A) Effect of alcohol extract on HCT116 cell viability; (B) Effect of alcohol extract on HGC27 cell viability; (C) Effect of BC alcohol extract on ROS levels in HCT116 cells; (D) Effect of CS alcohol extract on ROS levels in HCT116 cells; (E) Effect of BC alcohol extract on ROS levels in HGC27 cells; (F) Effect of CS alcohol extract on ROS levels in HGC27 cells. Asterisks indicate significant differences between groups (*** *P* < 0.001, ** *P* < 0.01, * *P* < 0.05).

For HGC27 cells (Fig. 9B), BC extract (125–4000 μg/mL) reduced viability from 72.53% to 11.14% (IC50 = 707.9 μg/mL), whereas CS extract (1000–12,000 μg/mL) reduced viability from 73.31% to 29.15% (IC50 = 1655 μg/mL). Both extracts inhibited HGC27 proliferation, with BC showing marginally higher efficacy; notably, both were more potent against HGC27 than HCT116.

Elevated ROS levels can induce DNA damage and apoptosis. In HCT116 cells (Fig. 9C–D), ROS levels declined with increasing extract concentrations yet remained above control levels. BC (250–1000 μg/mL) and CS (1250 μg/mL) significantly elevated intracellular ROS relative to the control. In HGC27 cells (Fig. 9E–F), ROS maintained dynamic equilibrium across concentrations; nevertheless, BC (250 and 500 μg/mL) and CS (125–2000 μg/mL) significantly increased ROS compared to the control. These findings suggest that BC and CS extracts may inhibit cancer cell growth, at least in part, through ROS-mediated mechanisms.

## 4 Discussion

The taste and volatile compounds of *A. cylindracea* are critical determinants of its market value, sales volume, and suitability for processed products (Liu, 2025). Previous study (Li et al., 2024) demonstrated that *A. cylindracea* cultivated on different substrates exhibited significant variations in nutritional composition, with crude polysaccharide, crude protein, and crude fiber contents ranging from 3.90% to 4.46%, 25.40% to 29.60%, and 7.75% to 9.15%, respectively. Similarly, (Yang et al., 2025) The soluble protein content of *A. cylindracea* declined from 95 mg/g to 60 mg/g following harvest. The results of the present study are generally consistent with these previous findings, although certain discrepancies were observed; these variations may be attributable to differences in cultivation substrate (Ahmed et al., 2024), extraction methodology (Karalti, 2026), and other experimental factors. Texture Profile Analysis (TPA) is an instrumental technique that employs a texture analyzer to simulate the cutting and chewing mechanics of the human oral cavity, thereby enabling the quantification of parameters including hardness, cohesiveness, springiness, gumminess, and chewiness. This approach can substantially minimize subjective bias in food quality evaluation and has been extensively applied to the textural assessment of diverse food products (Li et al., 2025). The hardness and chewiness of BC were significantly greater than those of CS, allowing BC to better resist mechanical stress during handling and processing, while demanding greater masticatory effort and delivering a richer mouthfeel.

In recent years, the integration of sensor technologies—such as electronic tongue and electronic nose systems—with chemometrics and machine learning algorithms has been widely adopted in food science research (Gil et al., 2025). Electronic tongue sensor responses are frequently correlated with the contents of taste-active compounds, including free amino acids and 5′-nucleotides (Zhu et al., 2022). Hou Z. et al. (Hou et al., 2022) investigated the effects of hot-air drying at different temperatures on the taste of *Suillus granulatus* and reported that drying at 60 °C produced significantly higher taste activity values for glutamic acid and 5′-guanylic acid, along with elevated equivalent umami concentration values and umami sensory scores from the electronic tongue, relative to other drying temperatures. The sourness, bitterness, sweetness, and umami of BC were significantly higher than those of CS. These results are consistent with our earlier findings on free amino acid content, 5′-nucleotide content, and equivalent umami concentration analysis, suggesting that BC possesses a richer umami profile and superior market competitiveness compared to CS. Beyond free amino acids and 5′-nucleotides, taste-active compounds in edible fungi also encompass organic acids, taste-active peptides, and other minor constituents. The amino acid addition-back results obtained in this study did not fully correspond to the taste evaluation results, possibly because the final taste perception of the samples is not governed by a single compound but rather by the synergistic and antagonistic interactions among various taste-active compounds (Sun et al., 2020; Yang et al., 2026).

Electronic nose and GC–MS techniques enable comprehensive evaluation of sample aroma profiles. Electronic tongue, electronic nose, and GC-IMS methodologies were employed to investigate the flavor characteristics of *Antrodia cinnamomea* cultivated under different conditions (Ma et al., 2025). Results from electronic nose and electronic tongue analyses indicated significant differences in the flavor characteristics of *A. cinnamomea* across the three culture methods; meanwhile, GC-IMS detected 75 volatile compounds, among which esters, alcohols, and ketones constituted the principal components. Numerous studies have demonstrated that the characteristic volatile compounds of edible fungi are predominantly eight‑carbon compounds, including 1-octen-3-ol, 3-octanol, (*E*)-2-octenal, and 1-octen-3-one. Among these, 1-octen-3-ol and 1-octen-3-one are regarded as signature aroma compounds across various edible fungi, conferring their distinctive mushroom-like odor (Selli et al., 2021). (Song et al., 2025) Qualitative and quantitative analyses of volatile compounds in *Pleurotus eryngii* cultivated by two different methods using SPME-GC–MS, identifying 15 volatile compounds as key aroma markers. Among these, 1-octen-3-one, 3-methylthiopropanol, isovaleraldehyde, methanethiol, and 3-octanone contributed most significantly to the overall aroma profile of *P. eryngii*. In the present study, 1-octen-3-one was identified as the most significant contributor to the aroma of both BC and CS, a finding consistent with previous reports. Nevertheless, variations in the contents of hexanal, 3-methylpentanal, 1-octen-3-ol, and 3-octanone accounted for the aroma distinctions between BC and CS.

In addition to basic nutrients, edible fungi contain a variety of bioactive compounds, including polyphenols, flavonoids, and polysaccharides, which enhance immune function and exert antibacterial, anticancer, and antioxidant activities (Rangel-Vargas et al., 2021). Cancer cells are highly sensitive to fluctuations in ROS levels and redox status; accordingly, both ROS elimination via scavenging enzymes and ROS elevation through enhanced production represent viable therapeutic strategies (Singh & Manna, 2022). (Hu et al., 2018) A polyphenol-rich extract was isolated from *P. eryngii*, identifying methyl gallate and catechin as key constituents. This extract induced cell cycle arrest and apoptosis in colon cancer cells, thereby suppressing the proliferation of human HCT116 colon cancer cells. (Shanmugapriya et al., 2017) The anticancer effects and mechanisms of *Calophyllum inophyllum* fruit extract against breast cancer cells were investigated, reporting that the extract induced apoptosis via mitochondrial pathways, as evidenced by elevated intracellular ROS levels, loss of mitochondrial membrane potential, and activation of caspase-3. The present study demonstrated that BC exerted significantly stronger inhibitory effects against both cancer cell lines than did CS, a difference potentially attributable to its higher polysaccharide content. Studies have elucidated the mechanism by which fucoglucogalactan from *A. cylindracea* induces lysosome-mediated apoptosis in colorectal cancer cells. This polysaccharide acts via an H3K27ac-regulated cathepsin D pathway, resulting in lysosomal dysfunction (Wang et al., 2023). A ubiquitin-like peptide purified from *A. cylindracea* acts via two pathways: stimulating nitric oxide (NO) production by macrophages and directly exerting antiproliferative effects (Ngai et al., 2003). The ethanol extracts of BC and CS induced apoptosis in cancer cells through ROS elevation. Notably, the decline in intracellular ROS levels in HCT116 cells at higher extract concentrations may reflect the activation of cellular antioxidant defense systems, which prevents ROS accumulation from surpassing the cytotoxic threshold (Brand et al., 2025). The superior anticancer activity of BC may arise from differences in its polysaccharide or protein content or structure, warranting future isolation of specific active components and elucidation of their mechanisms.

## 5 Conclusion

BC surpassed CS across all evaluated parameters. Nutritionally, BC contained significantly higher levels of crude polysaccharides, soluble protein, and crude fiber. Sensory analysis revealed BC required greater masticatory effort and offered a richer mouthfeel, with significantly elevated sourness, bitterness, sweetness, and umami. These taste differences were underpinned by higher total free amino acids (including umami, bitter, and sweet fractions), 5′-nucleotides, and EUC values in BC, with aspartic acid, glutamine, tryptophan, lysine, and 5′-cytidylic acid driving sourness, and glutamine and tryptophan driving umami. Volatile profiles also differed significantly between strains, with 1-octen-3-one being the predominant aroma-active compound in both. Additionally, BC exhibited stronger anticancer activity than CS, as evidenced by lower IC50 values against both cancer cell lines; both extracts induced cell death through elevated ROS levels. Owing to its high nutritional value and bioactivity, the BC has potential as a functional food and nutraceutical ingredient for use as an adjunct to cancer treatment. These findings provide a scientific basis for quality evaluation, functional development, and precision breeding of *A. cylindracea*, while elucidating relationships between quality traits and cultivation parameters to guide variety selection and management practices, and offer a scientific basis for the promotion of BC.

## CRediT authorship contribution statement

**Chengzhen Gu:** Writing – original draft, Data curation, Conceptualization. **Yangzheng Lin:** Validation, Supervision, Software. **Mengmeng Hao:** Visualization, Validation, Software, Data curation. **Zhiying Lin:** Software, Project administration, Methodology. **Jiaqi Fang:** Supervision, Resources, Project administration. **Jianwen Luo:** Resources, Methodology. **Shujing Sun:** Writing – review & editing.

## Declaration of competing interest

The authors declare that they have no known competing financial interests or personal relationships that could have appeared to influence the work reported in this paper.

## Data Availability

Data will be made available on request.
