# Supplementary material for: White agrocybe cylindracea outperforms the brown strain in nutritional value, flavor profile, and anticancer activity
Source: Food Chem X. 2026 Oct 5;39:104579. doi: 10.1016/j.fochx.2026.104579 (PMC13676911; doi:10.1016/j.fochx.2026.104579)
Supplement: Supplementary file 1 [file mmc1.docx]

Fig. S1. HPLC chromatograms of amino acids of BC and CS

Note: 1. Aspartic acid (Asp); 2. Glutamic acid (Glu); 3. Asparagine (Asn); 4. Histidine (His); 5. Glutamine (Gln); 6. Serine (Ser); 7. Arginine (Arg); 8. Threonine (Thr); 9. Proline (Pro); 10. Glycine (Gly); 11. Alanine (Ala); 12. Valine (Val); 13. Methionine (Met); 14. Tryptophan (Trp); 15. Leucine (Leu); 16. Phenylalanine (Phe); 17. Tyrosine (Tyr);18. Lysine (Lys).

Fig. S2. HPLC chromatograms of 5’-nucleotides of BC and CS

Note: 1. 5′-cytidylic acid (5′-CMP); 5′-uridylic acid (5′-UMP); 3. 5′-guanylic acid (5′-GMP); 4. 5′-xanthylic acid (5′-XMP); 5. 5′-adenylic acid (5′-AMP).
